# [4Fe-4S] cluster trafficking mediated by *Arabidopsis* mitochondrial ISCA and NFU proteins

**DOI:** 10.1074/jbc.RA120.015726

**Published:** 2021-01-13

**Authors:** Tamanna Azam, Jonathan Przybyla-Toscano, Florence Vignols, Jérémy Couturier, Nicolas Rouhier, Michael K. Johnson

**Affiliations:** 1 Department of Chemistry and Center for Metalloenzyme Studies, University of Georgia, Athens, Georgia, USA; 2 Université de Lorraine, INRAE, IAM, Nancy, France; 3 BPMP, Université de Montpellier, INRAE, CNRS, SupAgro, Montpellier, France

**Keywords:** mitochondria, iron-sulfur protein, *Arabidopsis thaliana*, protein-protein interaction, circular dichroism, Raman spectroscopy, iron-sulfur cluster trafficking, ISCA proteins, NFU proteins

## Abstract

Numerous iron-sulfur (Fe-S) proteins with diverse functions are present in the matrix and respiratory chain complexes of mitochondria. Although [4Fe-4S] clusters are the most common type of Fe-S cluster in mitochondria, the molecular mechanism of [4Fe-4S] cluster assembly and insertion into target proteins by the mitochondrial iron-sulfur cluster (ISC) maturation system is not well-understood. Here we report a detailed characterization of two late-acting Fe-S cluster-carrier proteins from *Arabidopsis thaliana*, NFU4 and NFU5. Yeast two-hybrid and bimolecular fluorescence complementation studies demonstrated interaction of both the NFU4 and NFU5 proteins with the ISCA class of Fe-S carrier proteins. Recombinant NFU4 and NFU5 were purified as apo-proteins after expression in *Escherichia coli*. *In vitro* Fe-S cluster reconstitution led to the insertion of one [4Fe-4S]^2+^ cluster per homodimer as determined by UV-visible absorption/CD, resonance Raman and EPR spectroscopy, and analytical studies. Cluster transfer reactions, monitored by UV-visible absorption and CD spectroscopy, showed that a [4Fe-4S]^2+^ cluster-bound ISCA1a/2 heterodimer is effective in transferring [4Fe-4S]^2+^ clusters to both NFU4 and NFU5 with negligible back reaction. In addition, [4Fe-4S]^2+^ cluster-bound ISCA1a/2, NFU4, and NFU5 were all found to be effective [4Fe-4S]^2+^ cluster donors for maturation of the mitochondrial apo-aconitase 2 as assessed by enzyme activity measurements. The results demonstrate rapid, unidirectional, and quantitative [4Fe-4S]^2+^ cluster transfer from ISCA1a/2 to NFU4 or NFU5 that further delineates their respective positions in the plant ISC machinery and their contributions to the maturation of client [4Fe-4S] cluster-containing proteins.

Iron-sulfur (Fe-S) clusters are protein cofactors that carry out crucial roles in diverse biological processes (1). In plants, these cofactors are present in proteins involved in photosynthesis, nitrogen and sulfur assimilation, respiration, and vitamin, DNA, and amino acid metabolism (2, 3). The most represented forms are the [4Fe-4S] and [2Fe-2S] clusters, with a few proteins incorporating cubane-type [3Fe-4S] clusters. Fe-S cluster biosynthesis and incorporation into client proteins is a regulated process implicating several dedicated assembly machineries, each involving multiple proteins. In plants, three machineries with a different subcellular distribution are present (2, 4). Whereas the plastidial sulfur utilization (SUF) machinery operates independently, the cytosolic iron-sulfur protein assembly machinery, required for both cytosolic and nuclear Fe-S proteins, depends on the mitochondrial iron-sulfur cluster (ISC) assembly machinery.

The molecular mechanisms for Fe-S cluster assembly are globally similar for each machinery even though the proteins involved may belong to different classes. As an example, the maturation of mitochondrial Fe-S proteins involves the orchestrated action of ∼20 proteins and can be divided into several sequential steps (5). The initial steps correspond to the assembly of a [2Fe-2S]^2+^ cluster on the Isu/ISCU/ISU scaffold protein. This *de novo* assembly involves a protein complex formed by the cysteine desulfurase Nfs1/NFS1 and two associated proteins, Isd11/ISD11 and Acp1/ACP1, which together supply the required sulfur atoms by catalyzing cysteine desulfuration (6, 7, 8, 9). An additional protein, frataxin (Yfh1/FH/FXN), is proposed to coordinate Fe^2+^ ion entry in the complex and to enhance the persulfide transfer reaction from NFS1 to U-type scaffold proteins (10, 11, 12, 13). A ferredoxin (Yah1/FDX) supplies the additional electrons required to reduce the persulfide to sulfide (13, 14). After assembly, the ISCU-bound [2Fe-2S]^2+^ cluster is conveyed and transferred using a chaperone/co-chaperone system to target the Fe-S cluster-ligating subunits of complex I and specific LYR motif-containing proteins such as SDHB, the Fe-S cluster-binding subunit of complex II, and LYRM7, an assembly factor for respiratory chain complex III as observed with human proteins (15). Another target for the holo-ISCU-chaperone/co-chaperone complex is the class II/monothiol glutaredoxin (GRX), namely Grx5 in yeast, GLRX5 in human, or GRXS15 in plants (16, 17). Grx5, which is the first acting Fe-S cluster transfer/carrier protein in yeast, can directly deliver [2Fe-2S] clusters to acceptor proteins, the identity of which remains to be determined. In addition, yeast Grx5 appears to be the last ISC protein connected to the cytosolic iron-sulfur protein assembly machinery (16, 18). Unlike in yeast, *Arabidopsis thaliana* (*At*, Arabidopsis) *grxs15* mutant lines are not viable, indicating that GRXS15 plays an essential, possibly different, role (19).

The biosynthesis of [4Fe-4S] clusters requires additional steps and another set of cluster transfer proteins, belonging to the A-type carrier (ATC), NFU, and MRP/NTPase protein classes. They are named Isa1/2 and ISCA1a/1b/2, and Nfu1 and NFU4/5, in *Saccharomyces cerevisiae* and *Arabidopsis*, respectively. The *Arabidopsis* P-loop NTPase member INDH is not found in *S. cerevisiae* because it is specific to the maturation of respiratory complex I, and it is also referred to as Ind1 in *Yarrowia lipolytica* or NUBPL in human (20, 21). In addition, two other types of maturation factors participate in these late assembly steps, namely Bol1/Bol3 and Iba57 in yeast or BOLA4 and IBA57.1 in *Arabidopsis*. Their precise roles are unclear so far, but BOLAs form complexes with GRXs and IBA57s with ISCAs (22, 23). *In vitro* studies using mammalian GLRX5 and ISCAs have suggested that the [2Fe-2S] to [4Fe-4S] cluster interconversion occurs during the Fe-S cluster transfer between the glutaredoxin and an ISCA1/2 heterodimer (24, 25). [4Fe-4S] cluster assembly or release from ISCA heterodimers may be facilitated by the BOLA and IBA57 maturation factors (26, 27). This conclusion is strengthened by the observation that in yeast and human, only [4Fe-4S] proteins are affected in *isa1/isca1*, *isa2/isca2*, and *iba57* mutants or cell lines (23, 28, 29, 30). So far, it has been demonstrated that INDH is connected to complex I of the respiratory chain, but the precise role and Fe-S cluster donor of INDH/Ind1/NUBPL remains to be determined (20, 21, 31, 32). The deletion of the unique mitochondrial Nfu1/NFU1 partially affects aconitase, lipoic acid synthase, and succinate dehydrogenase activities, indicating a nonessential role for Nfu1 as a [4Fe-4S]^2+^ cluster donor protein (33, 34, 35). Based on the specific or milder molecular phenotypes of the *nfu1* and *nubpl* mutants in yeast or human, it is assumed that these two types of transfer proteins act downstream of ATC proteins.

All representatives of the NFU family of proteins share a common NFU domain containing two conserved cysteines in a C*XX*C motif that binds an Fe-S cluster at the homodimer subunit interface. It can also be associated to an additional domain of variable nature, thus conferring a large diversity of structural organization among NFU proteins (36). There are five NFU proteins in *Arabidopsis* divided into two classes. NFU1/2/3 proteins are located in chloroplasts, whereas NFU4/5 are proposed to be located in the mitochondrial matrix (37). Plastidial NFU isoforms have an additional C-terminal NFU domain that does not have the Fe-S cluster-ligating cysteines, whereas NFU4/5 have an additional N-terminal domain of unknown function in the same region as found in mitochondrial Nfu1/NFU1 proteins from yeast and human. It is assumed that these extra domains are involved in targeting specific acceptor proteins, as was documented for the additional ATC domain present in *Escherichia coli* NfuA (36). So far, only plastidial-located NFUs have been characterized in plants. It was demonstrated that NFU2 and very likely NFU3 are required for the maturation of numerous [2Fe-2S]- and [4Fe-4S]-containing proteins in plants, including photosystem I subunits and a dihydroxyacid dehydratase involved in branched chain amino acid synthesis (38, 39, 40, 41). The importance of these proteins is indicated by the lethality of an *nfu2 nfu3* mutant in *Arabidopsis* (40). In contrast, NFU1 is dispensable, at least in standard growth conditions, and solely able to assemble and deliver [4Fe-4S] clusters (40, 42). The physiological role of the mitochondrial NFU4/5 isoforms has never been investigated *in planta* so far. However, both NFU4 and NFU5 are able to rescue the growth and biochemical phenotypes of the yeast *nfu1*Δ and *nfu1*Δ*isu1*Δ strains, suggesting that plant orthologs perform functions similar to yeast Nfu1 and are able to interact with the physiological partners of yeast Nfu1 (37, 43).

In the present study, we combined complementary *in vivo* and *in vitro* approaches to investigate the properties and interactions of *Arabidopsis* NFU4/5 proteins. The results demonstrate that NFU4 and NFU5 only bind [4Fe-4S] clusters, as is the case for the yeast and human mitochondrial counterparts. Among other late-acting ISC components, NFU4 and NFU5 interact with ISCA1 proteins, and we further showed that an ISCA1a/2 heterodimer is a competent Fe-S cluster donor for the mitochondrial NFU proteins in *Arabidopsis* and that [4Fe-4S]^2+^ cluster-bound ISCA1a/2, NFU4, and NFU5 are all competent for the maturation of mitochondrial apo-aconitase 2 (ACO2). This work clarifies the position of NFU4/5 proteins in the plant mitochondrial ISC machinery, acting directly downstream of ISCA proteins.

## Results

### What are the NFU4/5 partners among the ISC components?

**Figure 1:**
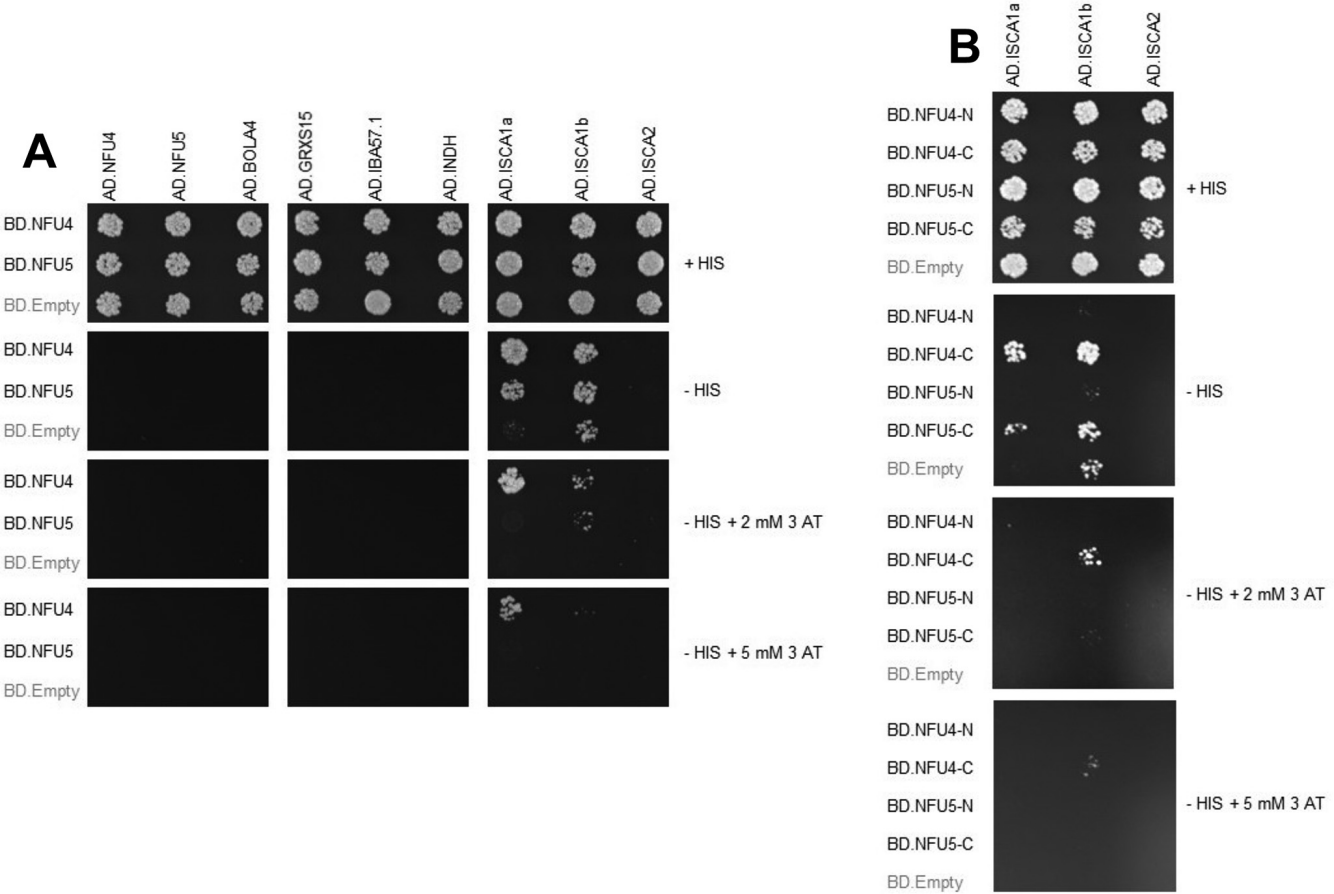
**Binary Y2H interaction between *Arabidopsis* NFU4 and NFU5 and other late-acting ISC components.***A*, the yeast strain CY306 was cotransformed with sequences encoding the mature forms of NFU4 and NFU5 proteins fused at the C terminus of the Gal4 DNA-binding domain (*BD*) as indicated and other ISC components fused at the C terminus of the Gal4 activation domain (*AD*). The cotransformed yeast cells were plated on a control medium containing histidine (+ HIS) and interactions were tested on medium without histidine (− HIS) in the presence of 2 mm or 5 mm 3-AT as indicated. Yeast growth was analyzed after 5 days. Only ISCA1b exhibited a slight autoactivation in the – His medium which disappeared in the presence of 3-AT. No autoactivation was observed for BD-NFU4 and NFU5 (not shown). *B*, the yeast strain CY306 was cotransformed with sequences encoding the N- or C-terminal domains of NFU4 and NFU5 proteins fused at the C terminus of the Gal4 DNA-binding domain (*BD*) and ISCA1a or ISCA1b fused at the C terminus of the Gal4 activation domain (*AD*). The NFU4-N and -C domains correspond respectively to amino acids 80–168 and 187–283 of NFU4, whereas the NFU5-N and -C domains correspond respectively to amino acids 75–163 and 182–275 of NFU5. The conditions are as in (*A*).

To identify the partners of mitochondrial NFU proteins among ISC components, we tested whether NFU4 and NFU5 physically interacted with ISC components involved in the late steps, starting from GRXS15, using binary yeast two-hybrid (Y2H). Only the coexpression of NFU4 or NFU5 with ISCA1a or ISCA1b allowed yeast cell growth in absence of histidine, indicating an interaction between these transfer proteins (Fig. 1*A*). These interactions were only detectable when NFU4/5 were fused to the DNA-binding domain of Gal4. To determine whether both domains of NFU4/5 proteins are necessary for interaction and potentially to assign a role to the additional N-terminal domain, both NFU4/NFU5 domains have been separately expressed in fusion with the Gal4 DNA-binding domain and tested with ISCA1a/b, because interactions were only observed with this combination using mature proteins. The N-terminal domain comprised amino acids 80–168 for NFU4 and 75–163 for NFU5, whereas the C-terminal NFU domain harboring the conserved C*XX*C motif retained amino acids 187–283 for NFU4 and 182–275 for NFU5. Only yeast cells coexpressing ISCA1a/b and the NFU domain of NFU4 or NFU5 grew on medium lacking histidine, indicating that this domain is mostly responsible for these interactions (Fig. 1*B*).

**Figure 2:**
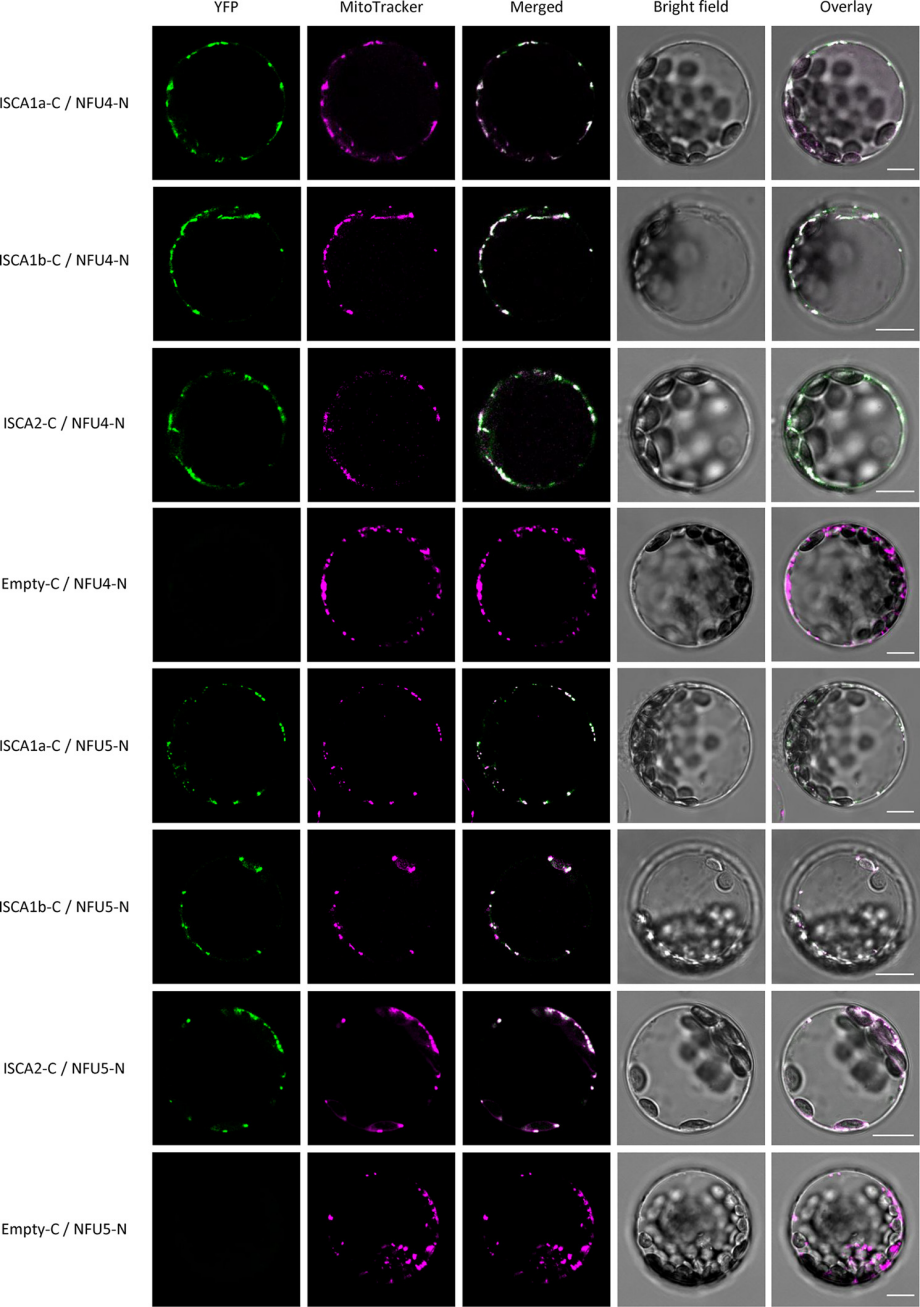
**BiFC interactions between *Arabidopsis* mitochondrial ISCAs and NFUs.***Arabidopsis* protoplasts isolated from 4-week-old plantlets were transfected with combinations of two vectors expressing either NFU4 or NFU5 fused to the N-terminal region of the YFP protein (*NFUs-N* in *panels*) and ISCAs fused to the C-terminal region of YFP (*ISCA-C* in *panels*). The YFP fluorescence was recorded 24 h posttransfection by confocal microscopy. Negative controls verifying that none of the NFU proteins tested alone with the empty partner vector can restore YFP fluorescence are shown as *Empty-C/NFUs-N* in *panels*; those verifying ISCA-C/Empty-N combinations are shown in Fig. S1. Protoplast cotransfections using opposite protein chimera (ISCA-N with NFUs-C) provided similar patterns but also showed a strong tendency to form cytosolic aggregates (not shown). All images were captured using the LAS X software at confocal plans at selected Z dimensions and processed using the Adobe Photoshop software. Results are representative of three independent bombardment experiments including the analysis of 10–20 cells per transformation event. MitoTracker® Orange CMXRos (Invitrogen) was used at 100 nm to label mitochondria within cells. *Bars* = 10 µm.

To validate the interaction between NFU4 or NFU5 and ISCA proteins in a plant-based reporter system, bimolecular fluorescence complementation (BiFC) experiments were performed in *Arabidopsis* protoplasts. A reconstituted YFP fluorescence signal colocalizing with the mitochondrial marker was observed when NFU4 or NFU5 were coexpressed with ISCA1a, and ISCA1b in accord with Y2H results, but also with ISCA2 (Fig. 2 and Fig. S1).We have two possible explanations for the divergence of the results obtained by Y2H and BiFC for ISCA2. Either there is indeed a direct interaction between NFU4/5 and ISCA2 that cannot be detected by Y2H, or the BiFC signal is due to the presence of ISCA1a/b, which allows forming a ternary complex in plant cells. Altogether, these results indicate that both NFU4 and NFU5 proteins are able to form a stable complex with ISCAs (at least with ISCA1s) and that these interactions occur in plant mitochondria.

### Nature and properties of cluster-bound forms of At NFU4 and NFU5

**Figure 3:**
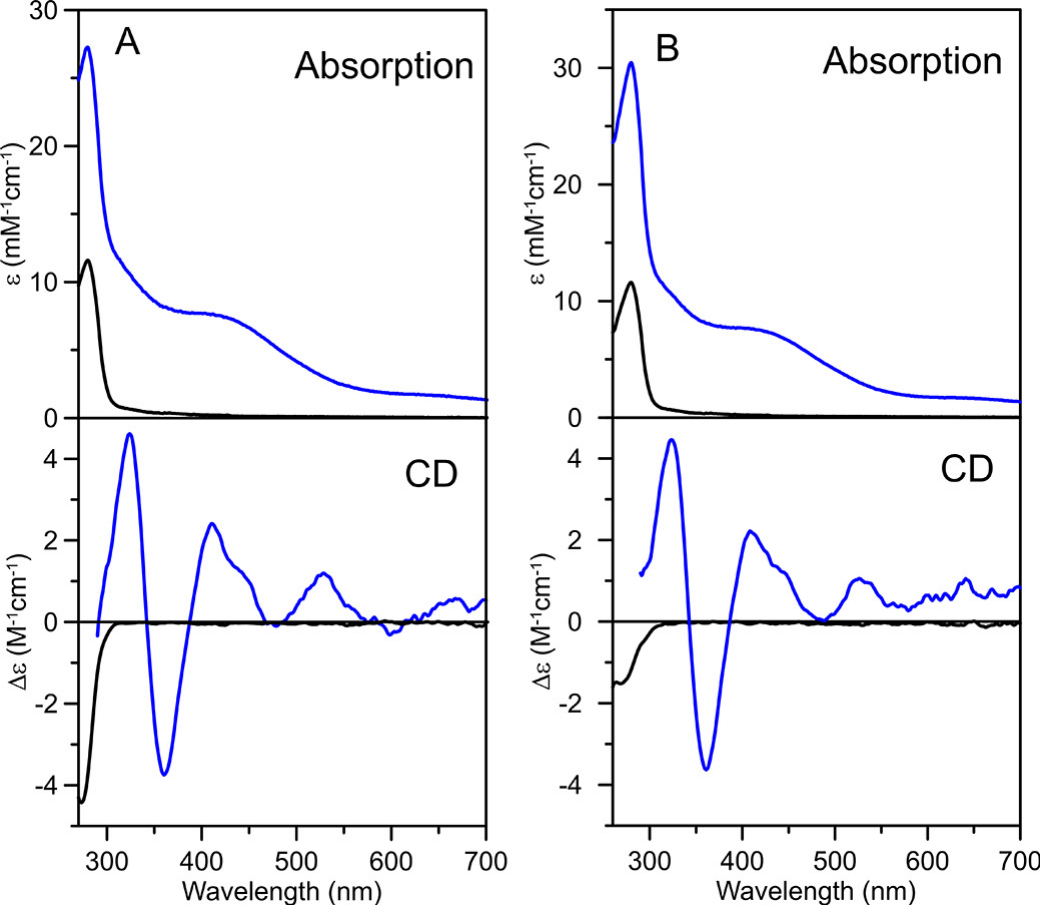
**Room temperature UV-visible absorption spectra and CD spectra of *At* NFU4 and *At* NFU5.** As-purified apo-NFU4 and NFU5 are shown as *black lines* and reconstituted NFU4 (*A*) and NFU5 (*B*) are shown as *blue lines*. All ε and Δε values are based on NFU4 and NFU5 protein monomer concentration.

The nature of Fe-S clusters assembled on NFU4 and NFU5 was determined through spectroscopic and analytical studies of reconstituted samples. Aerobically purified NFU4 and NFU5 have no visible absorption, indicating that they were purified as apo-proteins (Fig. 3). Anaerobic cysteine desulfurase-mediated cluster reconstitution experiments were conducted in the presence of DTT to address the ability of NFU4 and NFU5 to incorporate Fe-S clusters. Reconstituted NFU4 and NFU5 eluted from the Q-Sepharose column as a single brown fraction. The UV-visible absorption spectra of reconstituted NFU4 and NFU5 are very similar, comprising broad shoulders centered near 320 and 410 nm, which are characteristic of [4Fe-4S]^2+^ clusters (Fig. 3, *blue lines*). The UV-visible CD spectra of reconstituted NFU4 and NFU5 are also very similar, with intense positive and negative bands at 310 and 350 nm, respectively, and weaker positive bands at 440 and 510 nm. These CD spectra are very similar to the reconstituted [4Fe-4S]^2+^ cluster-bound form of chloroplast NFU1 (42) but quite different from the reconstituted [4Fe-4S]^2+^ cluster-bound form of chloroplast NFU2 (38), suggesting at least two distinct classes of plant NFU proteins. The broad shoulder in the absorption spectra centered at 400 nm (*A*_400_/*A*_280_ = 0.28 ± 0.02) with a molar extinction coefficient ε_400_ = 7.5 ± 0.5 mm^−1^cm^−1^, based on NFU monomer concentration, is indicative of approximately one [4Fe-4S]^2+^ cluster per NFU4 and NFU5 dimer, in accordance with the protein and iron analyses indicating 2.2 ± 0.3 Fe per NFU4/5 monomer (44, 45, 46).

**Figure 4:**
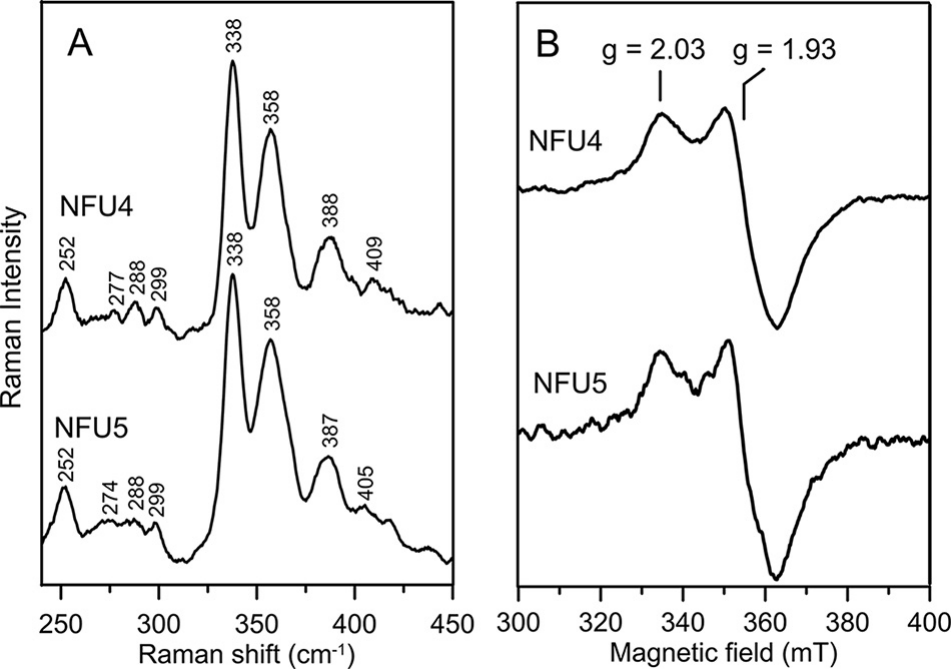
**Comparison of the resonance Raman and EPR spectra of reconstituted *At* NFU4 and *At* NFU5.***A*, resonance Raman spectra of reconstituted NFU4 and NFU5 recorded at 17 K with 457.9-nm excitation. Each spectrum is the sum of 100 individual scans, with each scan involving photon counting for 1 s at 0.5-cm^−1^ increments, with 7-cm^−1^ spectral resolution. Bands due to frozen buffer solution have been subtracted from both spectra. *B*, X-band EPR spectra of reconstituted NFU4 and NFU5 reduced with one reducing equivalent of dithionite and frozen immediately in liquid nitrogen. EPR conditions: microwave frequency, 9.60 GHz; microwave power, 10 milliwatt; modulation amplitude, 0.63 mT; temperature, 10 K.

The vibrational properties of the [4Fe-4S]^2+^ cluster in NFU4 and NFU5 were investigated by low temperature resonance Raman spectroscopy. The resonance Raman spectra of NFU4 and NFU5, shown in Fig. 4*A*, are uniquely characteristic of a [4Fe-4S]^2+^ cluster. The spectra are similar in terms of Fe-S stretching frequencies and relative intensities to those reported for all-cysteinyl-ligated [4Fe-4S]^2+^ clusters in ferredoxins and the nitrogenase Fe protein, see Table 1 (48, 68). The [4Fe-4S]^2+^ clusters in NFU4/5 and the nitrogenase Fe protein are both ligated at the subunit interface via C*XX*C motifs in each subunit. The Fe-S stretching modes are readily assigned, based on normal mode calculations and ^34^S^b^/^32^S^b^ isotope shifts reported for cubane [Fe_4_S^b^_4_]S^t^_4_ units in ferredoxins and appropriate analog complexes, under idealized *T_d_* symmetry (see Table 1) (45, 47, 48). The most intense bands in the spectra of NFU4 and NFU5 are the totally symmetric (A_1_) breathing modes of the cubane [Fe_4_S^b^_4_] core, which are both observed at 338 cm^−1^. This is in accord with all-cysteinyl ligation (frequency range 333–339 cm^−1^), whereas replacement of one ligated cysteine by hydroxide, serinate, or aspartate generally results in higher frequencies (frequency range 340–343 cm^−1^) (49).

**Table 1:** Fe-S stretching frequencies (cm^−1^) and vibrational assignments for the [4Fe-4S]^2+^ centers in *Clostridium pasterianum* 8Fe Fd, *C. pasterianum* N_2_ase Fe protein, *A. thaliana* NFU4, and *A. thaliana* NFU5

Assignments under *T_d_* symmetry^a^	*C. pasterianum* 8Fe ferredoxin	*C. pasterianum* N_2_ase Fe protein	*A. thaliana* NFU4	*A. thaliana* NFU5
**Mainly terminal ν(Fe-S^t^)**				
A_1_	395	391	409 or 388	405 or 387
T_2_	363, 351	356	358	358
**Mainly bridging ν(Fe-S)**				
T_2_	380	391	388	387
A_1_	338	335	338	338
E	298, 276	281	299, 288	299, 288
T_1_	276, 266	265	288, 277	288, 274
T_2_	251	248	252	252


a Symmetry labels assuming idealized *T_d_* symmetry for the Fe_4_S_4_^b^S_4_^t^ core, where Fe-S^b^ and Fe-S^t^ indicate bridging and terminal stretching, respectively.

Reduction of [4Fe-4S]^2+^ cluster-containing NFU4 and NFU5 by incubation with a 10-fold excess of dithionite for 10 min resulted in complete cluster degradation based on UV-visible absorption and EPR studies. This indicates that the reduced [4Fe-4S]^1+^ clusters are not stable in NFU4 and NFU5 and are unlikely to be physiologically relevant. This conclusion is supported by the observation of EPR signals indicative of a reduced [4Fe-4S]^1+^ cluster in samples of reconstituted NFU4 and NFU5 reduced with one reducing equivalent of dithionite and frozen within 3 s in liquid nitrogen (Fig. 4*B*). Identical, near-axial, fast-relaxing *S* = 1/2 EPR signals with *g*_||_ = 2.03 and *g*_⊥_ = 1.93, maximally accounting for 0.35 spins per NFU dimer, were observed for both NFU4 and NFU5. These resonances were only observable without broadening below 30 K, indicating fast relaxation, which is characteristic of [4Fe-4S]^1+^ clusters. The low *S* = 1/2 spin quantification does not result from mixed spin [4Fe-4S]^1+^ clusters, because low-field resonances around *g* = 5 indicative of *S* = 3/2 [4Fe-4S]^1+^ clusters were not observed. Rather, the low spin quantification appears to be a consequence of the [4Fe-4S]^1+^ cluster being a transient intermediate in the reductive cluster degradation pathway, because increasing the reaction time before freezing resulted in progressively decreasing spin quantification.

### Incorporation of clusters on NFU4 and NFU5 via cluster transfer from ISCA1a/2

**Figure 5:**
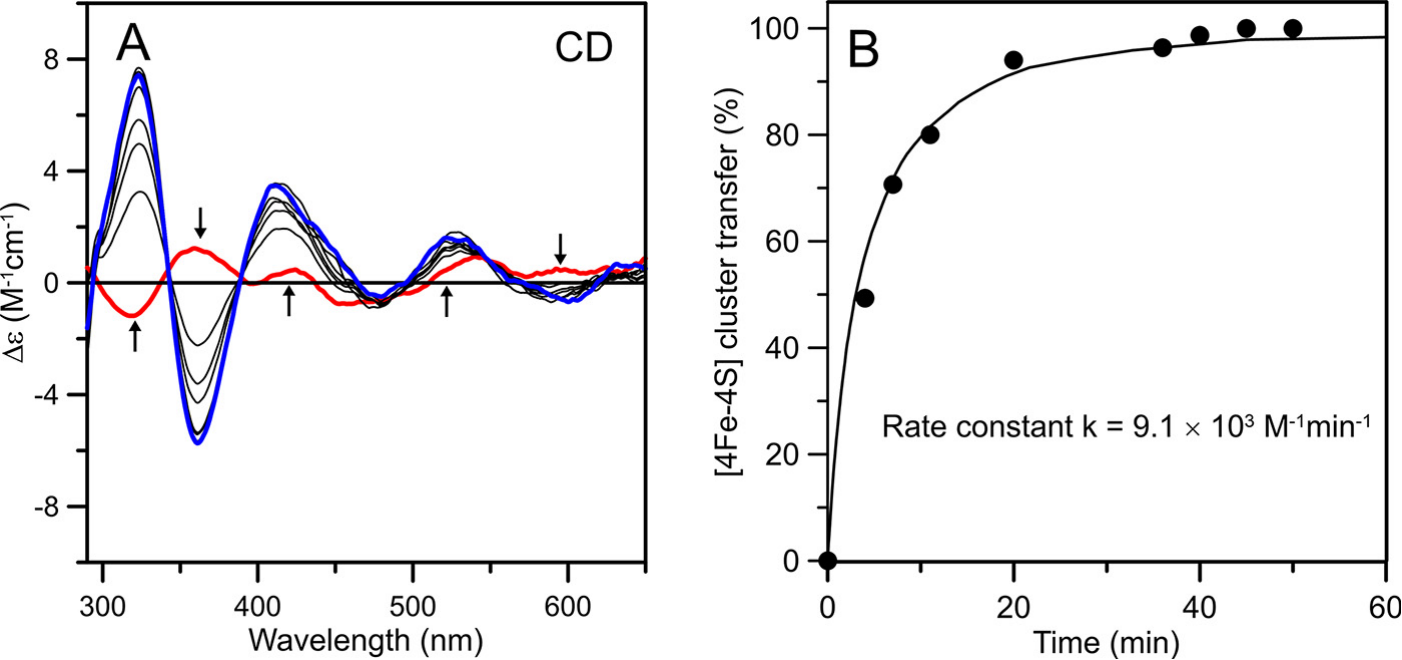
**Cluster transfer from *At* [4Fe-4S]^2+^-ISCA1a/2 to *At* apo-NFU4 monitored by CD spectroscopy as a function of time.***A*, CD spectra of the cluster transfer reaction mixture that was initially 30 µm in ISCA1a/2 [4Fe-4S]^2+^ clusters and 60 µm in DTT-pretreated apo-NFU4 monomer. The *thick red line* corresponds to [4Fe-4S]^2+^-ISCA1a/2 recorded before addition of apo-NFU4 to the reaction mixture. The *thin gray lines* correspond to CD spectra recorded at 4, 7, 11, 20, 36, 40, 45, and 50 min after the addition of apo-NFU4. The *thick blue line* corresponds to complete [4Fe-4S]^2+^ cluster transfer to NFU4. The *arrows* indicate the direction of intensity change with increasing time at selected wavelengths and Δε values were calculated based on the initial concentration of [4Fe-4S]^2+^ clusters in the reaction mixture. The cluster transfer reaction was carried out under anaerobic conditions at room temperature in 100 mm Tris-HCl buffer at pH 7.8. *B*, kinetic simulation of cluster transfer from [4Fe-4S]^2+^-ISCA1a/2 to apo-NFU4 based on second-order kinetics and the initial concentrations of [4Fe-4S]^2+^ clusters on [4Fe-4S]^2+^-ISCA1a/2 and of apo-NFU4. Percent cluster transfer was assessed by the difference in CD intensity at 326 and 362 nm (*black circles*) and simulated with a second-order rate constant of 9.1 × 10^3^m^−1^ min^−1^ (*black line*). The residual [2Fe-2S]^2+^-ISCA1a/2 peak-to-trough CD intensity at 362 and 326 nm at zero time was added on to each data point as there is no evidence for any [2Fe-2S]^2+^ cluster transfer.

**Figure 6:**
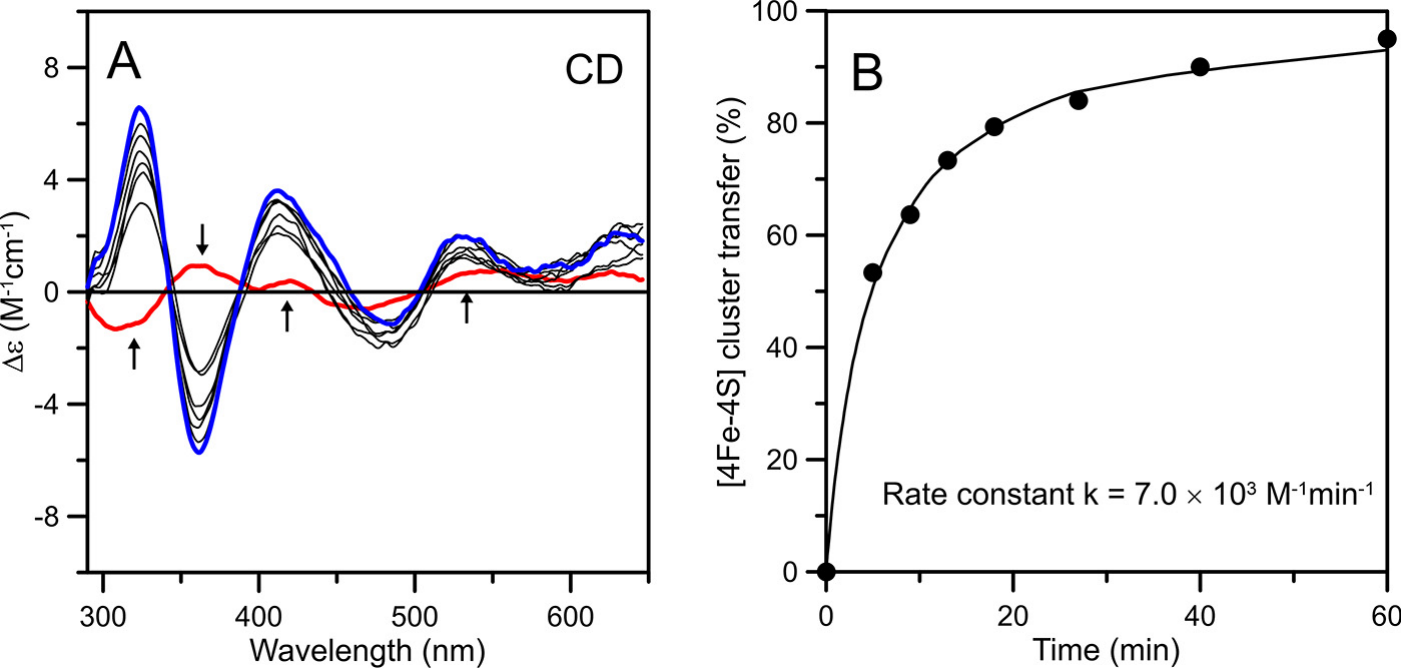
**Cluster transfer from *At* [4Fe-4S]^2+^-ISCA1a/2 to *At* apo-NFU5 monitored by CD spectroscopy as a function of time.***A*, CD spectra of the cluster transfer reaction mixture that was initially 40 µm in ISCA1a/2 [4Fe-4S]^2+^ clusters and 80 µm in DTT-pretreated apo-NFU5 monomer. The *thick red line* corresponds to [4Fe-4S]^2+^-ISCA1a/2 recorded before addition of apo-NFU5 to the reaction mixture. The *thin gray lines* correspond to CD spectra recorded at 5, 9, 13, 18, 27, 40, and 60 min after the addition of apo-NFU5. The *thick blue line* corresponds to complete [4Fe-4S]^2+^ cluster transfer to NFU5. The *arrows* indicate the direction of intensity change with increasing time at selected wavelengths, and Δε values were calculated based on the initial concentration of [4Fe-4S]^2+^ clusters in the reaction mixture. The cluster transfer reaction was carried out under anaerobic conditions at room temperature in 100 mm Tris-HCl buffer at pH 7.8. *B*, kinetic simulation of cluster transfer from [4Fe-4S]^2+^-ISCA1a/2 to apo-NFU5 based on second-order kinetics and the initial concentrations of [4Fe-4S]^2+^ clusters on [4Fe-4S]^2+^-ISCA1a/2 and of apo-NFU5. Percent cluster transfer was assessed by the difference in CD intensity at 326 and 362 nm (*black circles*) and simulated with a second-order rate constant of 7.0 × 10^3^m^−1^ min^−1^ (*black dots*). The residual [2Fe-2S]^2+^-ISCA1a/2 peak-to-trough CD intensity at 362 and 326 nm at zero time was added on to each data point, because there is no evidence for any [2Fe-2S]^2+^ cluster transfer.

Cluster transfer experiments involving [2Fe-2S]^2+^-GRXS15 and both as-purified [2Fe-2S]^2+^ and reconstituted [4Fe-4S]^2+^ cluster-bound forms of ISCA1a/2 were carried out to assess the cluster donor for NFU4 and NFU5. No cluster transfer was observed using either [2Fe-2S]^2+^-ISCA1a/2 or [2Fe-2S]^2+^-GRXS15 as cluster donors for apo NFU4/5, as evidenced by the unchanged [2Fe-2S]^2+^-ISCA1a/2 or [2Fe-2S]^2+^-GRXS15 CD spectra for 30 min after addition of a 2-fold excess of apo-NFU4/5, data not shown. In contrast, [4Fe-4S]^2+^ cluster transfer from ISCA1a/2 was shown to be effective for incorporating [4Fe-4S]^2+^ clusters in both NFU4 and NFU5 (Figure 5, Figure 6). Reconstituted ISCA1a/2 containing 25% [2Fe-2S]^2+^-ISCA1a/2 and 75% [4Fe-4S]^2+^-ISCA1a/2 was used as the donor. Because [4Fe-4S]^2+^-ISCA1a/2 exhibits negligible visible CD, the CD spectrum of the donor arises solely from [2Fe-2S]^2+^-ISCA1a/2, which remains unchanged during [4Fe-4S]^2+^ cluster transfer, because the [2Fe-2S]^2+^ cluster on ISCA1a/2 cannot be transferred to NFU4 or NFU5. Anaerobic [4Fe-4S]^2+^ cluster transfer from reconstituted ISCA1a/2 to DTT-pretreated apo-NFU4 and -NFU5 with a 1:1 donor:acceptor ratio was 80–90% complete after 20 min (Figure 5, Figure 6). Percent cluster transfer was assessed by the difference in CD intensity at 326 and 362 nm (corrected for the contribution from [2Fe-2S]^2+^-ISCA1a/2). Best fits to second-order kinetics yielded a rate constant of 9.1 ± 0.9 × 10^3^m^−1^ min^−1^ for NFU4 and 7.0 ± 0.7 × 10^3^m^−1^ min^−1^ for NFU5, based on the initial concentrations of [4Fe-4S]^2+^ clusters on ISCA1a/2 and the apo-NFU4/5 dimers. Control studies showed no reaction for the reverse cluster transfer (see Fig. S2), indicating a unidirectional reaction, and absorption studies showed no degradation of the [4Fe-4S]^2+^ cluster on ISCA1a/2 in the absence of NFU4 or NFU5 over the time course of the reaction, indicating intact cluster transfer.

In summary, we conclude that the rapid, quantitative, and unidirectional cluster transfer from [4Fe-4S]^2+^-ISCA1a/2, rather than NFU4 or NFU5, is likely to be a physiologically relevant pathway in plants. In addition, the results indicate that ISCA1a/2, rather than NFU4 or NFU5, is responsible for [2Fe-2S]^2+^ to [4Fe-4S]^2+^ cluster conversions in late stages of mitochondrial Fe-S cluster biogenesis and that NFU4 and NFU5 are obligate [4Fe-4S]^2+^ cluster trafficking proteins.

### Activation of At ACO2 using cluster-loaded forms of At ISCA1a/2, NFU4, and NFU5

**Figure 7:**
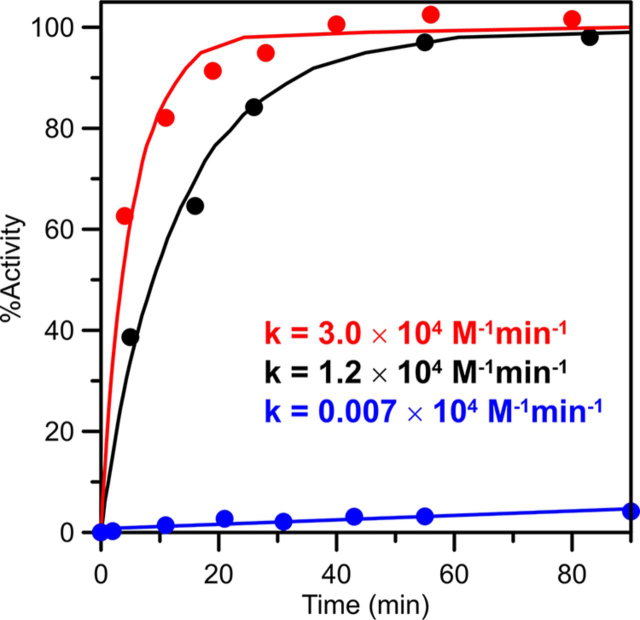
**Activation of apo-ACO2 using [4Fe-4S] cluster-bound ISCA1a/2, [4Fe-4S] cluster-bound NFU5, and [2Fe-2S] cluster-bound ISCA1a/2.** Apo-ACO2 (2.4 µm) was incubated with [4Fe-4S] cluster-loaded ISCA1a/2 (*red data*) or NFU5 (*black data*) (both 7.4 µm in [4Fe-4S] clusters) and [2Fe-2S] cluster-loaded ISCA1a/2 (*blue data*) (14.5 µm in [2Fe-2S]^2+^ clusters) at room temperature under anaerobic conditions. 10-µl aliquots of the reaction mixture were removed at selected time points and assayed immediately for aconitase activity. Residual aconitase activity of apo-ACO2, in the absence of a cluster donor, was assessed and subtracted from all measured activities. Aconitase specific activity as a function of incubation time with the cluster donor was expressed as a percentage of the maximal specific activity of [4Fe-4S]^2+^ cluster-replete ACO2. *Solid lines* are best fits to second-order kinetics, with the indicated rate constants, k, based on the initial concentrations of apo-ACO2 and [4Fe-4S]^2+^ clusters on ISCA1a/2 or NFU5 and half the initial [2Fe-2S]^2+^ cluster concentration of [2Fe-2S]-ISCA1a/2.

The ability of [4Fe-4S] cluster-loaded forms of ISCA1a/2, NFU4 and NFU5, and [2Fe-2S] cluster-loaded ISCA1a/2 to promote maturation of apo mitochondrial aconitase, ACO2, was assessed by monitoring aconitase activity as a function of time, after addition of a 3-fold excess of [4Fe-4S]^2+^ clusters or a 6-fold excess of [2Fe-2S]^2+^ clusters. Only the [4Fe-4S] cluster donors were effective in rapid restoration of ACO2 activity, with second-order rate constants of 3.0 ± 0.3 × 10^4^m^−1^ min^−1^ for [4Fe-4S] cluster transfer from ISCA1a/2 and 1.2 ± 0.3 × 10^4^m^−1^ min^−1^ for [4Fe-4S] cluster transfer from NFU4 and NFU5 (Fig. 7). Because of the similarity of results obtained for [4Fe-4S]-NFU4 and [4Fe-4S]-NFU5, only NFU5 data are shown. Both reactions are at least 10× faster than those observed under the same conditions with equivalent amounts of Fe^2+^ and S^2-^ ions, indicating that both are a consequence of intact cluster transfer rather than cluster degradation and reassembly on ACO2. The negligible rate of restoration of aconitase activity using [2Fe-2S]-ISCA1a/2 indicates that consecutive [2Fe-2S]^2+^ cluster transfers followed by *in situ* two-electron reductive coupling is not a viable mechanism for maturation of the aconitase [4Fe-4S] cluster.

## Discussion

In mitochondria, the maturation of Fe-S proteins depends on the ISC machinery. Unlike the *de novo* assembly of a [2Fe-2S] cluster on Isu/ISCU, the molecular mechanisms involved in cluster conversion, trafficking, and insertion in client proteins are less well-characterized. In this work, we provide new insights into the biochemical properties and roles of mitochondrial NFU4 and NFU5 in the plant ISC machinery.

NFU proteins have emerged as a major class of Fe-S cluster-carrier proteins capable of binding and trafficking [2Fe-2S]^2+^ or [4Fe-4S]^2+^ clusters that are bound by conserved C*XX*C motifs at the subunit interface of a homodimer. This is well-illustrated by *Helicobacter pylori* Nfu (50) and by our previous work on *Arabidopsis* chloroplastic NFU2, which both purify as [2Fe-2S]^2+^ cluster-containing recombinant proteins when expressed in *E. coli* and can be obtained in either a [2Fe-2S]^2+^ or [4Fe-4S]^2+^ cluster-bound form *via* anaerobic reconstitution of the apo-proteins (38). [2Fe-2S]-NFU2 has been shown to be a competent [2Fe-2S]^2+^ cluster donor for GRXS16 and chloroplast ferredoxin (38, 51), whereas [4Fe-4S]-NFU2 was shown to be the likely physiological [4Fe-4S]^2+^ cluster donor for adenosine 5′-phosphosulfate reductase (38). However, as discussed below, [2Fe-2S] cluster trafficking by NFU proteins may be restricted to the SUF systems for Fe-S cluster assembly in bacteria and plastids, which function under higher levels of O_2_.

In contrast, the human and yeast mitochondrial NFU proteins are part of the ISC machinery and have been proposed to function exclusively as [4Fe-4S]^2+^ cluster-carrier proteins based on both *in vivo* and *in vitro* evidence (33, 34, 35, 52, 53, 54). This hypothesis has recently been challenged in a series of five publications by Cowan and co-workers (55, 56, 57, 58, 59), which claim that human mitochondrial NFU1 is a [2Fe-2S]^2+^ cluster-carrier protein, based on UV-visible absorption/CD and EPR data. This interpretation is incorrect, based on our characterization of the homologous mitochondrial *At* NFU4 and NFU5 as [4Fe-4S]^2+^ cluster-carrier proteins. The UV-visible absorption and CD spectra of reconstituted NFU4 and NFU5 are essentially the same as those reported for reconstituted human NFU1 (55). However, the resonance Raman spectra of reconstituted NFU4 and NFU5 and the EPR spectra of reduced samples unambiguously demonstrate that these absorption and CD attributes are indicative of a [4Fe-4S]^2+^ cluster, not a [2Fe-2S]^2+^ cluster. Moreover, Mössbauer and NMR studies have also demonstrated that reconstituted human NFU1 exclusively contains a [4Fe-4S]^2+^ cluster (53, 54). This reinterpretation necessitates a major reevaluation of the results and conclusions made by Cowan and co-workers (55, 56, 57, 58, 59) about human NFU1. The discovery that both NFU4 and NFU5 are [4Fe-4S]^2+^ cluster-binding proteins, coupled with our inability to assemble a [2Fe-2S]^2+^ cluster on NFU4 and NFU5 either by reconstitution or cluster transfer from [2Fe-2S]-ISCA1a/2 or [2Fe-2S]-GRXS15, is in accord with the statement that all mitochondrial NFU proteins function in [4Fe-4S]^2+^ cluster trafficking. Nevertheless, we cannot rule out the possibility that [4Fe-4S]^2+^ cluster assembly on NFU4/5 in plants can also occur via [2Fe-2S]^2+^ cluster transfer from a GRXS15/BOLA4 complex, as recently proposed for the formation of human mitochondrial [4Fe-4S]-NFU1 *via* [2Fe-2S]^2+^ clusters provided by a GLRX5/BOLA3 complex (60).

The identification of NFU4/5 partners among the late-acting ISC components was unexplored in plants prior to this work. We obtained robust data demonstrating a specific and direct physical interaction with ISCA1 isoforms. The observation of intact [4Fe-4S] cluster transfer from ISCA1a/2 to NFU4/5 validates this interaction and suggests that in such heterodimers, ISCA1 isoforms are likely responsible for NFU4/5 recruitment. An interaction between mitochondrial NFU and ISCA1 proteins has been also observed but not characterized in detail in other organisms. In pulldown experiments, only mouse ISCA1, not ISCA2, retained NFU1 (30). In contrast, pulldown experiments using yeast Nfu1 allowed isolation of both Isa1 and Isa2 (35). It is possible that Isa2 has been detected because of a complex with Isa1 as we proposed to explain the positive BiFC result obtained with ISCA2. The NFU-ATC interaction seems largely conserved in evolution. In fact, *E. coli* NfuA interacts with all three ATC proteins (IscA, SufA, and ErpA), and the *Arabidopsis* chloroplastic NFU1-SUFA1 couple also forms a complex (36, 42). Considering the differences in NFU domain organization, such conservation suggests that only the NFU domain is crucial for the interaction with ATCs. This is what we observed by Y2H because the NFU domain of NFU4/5 was sufficient to form a complex with ISCA1 proteins. However, the opposite was observed for *E. coli* NfuA, because the N-terminal ATC domain, which lacks conserved cysteine residues, was shown to promote interaction of NfuA with its target proteins (36). Hence, the molecular function of the additional N-terminal domain found in mitochondrial NFUs remains to be further characterized. The possibilities include roles in recruiting some specific [4Fe-4S] cluster-acceptor proteins, stabilizing the interactions once formed, or promoting complex dissociation after Fe-S cluster transfer.

Cluster transfer studies also implicate that ISCA proteins act upstream of NFUs because only a unidirectional transfer from [4Fe-4S]-ISCA1a/2 to apo-NFU4 and -NFU5 was observed. The second-order rate constants for intact [4Fe-4S]^2+^ cluster transfer from [4Fe-4S]-ISCA1a/2 to NFU4 and NFU5, 9.1 ± 0.9 × 10^3^m^−1^ min^−1^ and 7.0 ± 0.7 × 10^3^m^−1^ min^−1^, respectively, are potentially physiologically relevant. However, these cluster transfers were obtained in the absence of mitochondrial IBA57.1. Previous results with human ISCA1/2 (25) argue against IBA57 playing a role in [4Fe-4S]^2+^ cluster assembly on ISCA1/2 heterodimers. Nevertheless, Iba57 has been shown to form a complex with Isa1/2 in yeast mitochondria, and depletion of any of these proteins in human and yeast cells resulted in decreased activities of key [4Fe-4S] cluster-containing enzymes (28, 29). Therefore, IBA57 may be required for preventing oxidative degradation of the assembled [4Fe-4S]^2+^ cluster and/or facilitating [4Fe-4S]^2+^ cluster transfer to client proteins. Alternatively, recent *in vitro* studies of human proteins have shown the formation of an ISCA2-IBA57 heterocomplex with a bridging [2Fe-2S] cluster that is resistant to oxidative degradation and is capable of activating aconitase (27). Unfortunately, our inability thus far to express IBA57.1 as a soluble protein has precluded *in vitro* experiments to investigate its role in plant mitochondrial Fe-S cluster biogenesis.

In light of the nonessential role of NFU1 in yeast and human mitochondria, as evidenced by partially defective Fe-S enzymes aconitase, succinate dehydrogenase, and lipoic acid synthase in cells lacking NFU1 (33, 34, 35, 61), we asked whether *Arabidopsis* ISCA1a/2 heterodimers function solely as [4Fe-4S]^2+^ cluster assembler proteins that supply downstream [4Fe-4S]^2+^ cluster-carrier proteins such as NFU4/5 or whether they also function in terminal [4Fe-4S]^2+^ cluster delivery to a subset of client enzymes. The *in vitro* cluster transfer results presented in this work suggest that the latter is correct, based on the ability of [4Fe-4S]-ISCA1a/2 to effect rapid maturation of ACO2 *via* intact cluster transfer (second-order rate constant of 3.0 ± 0.3 × 10^4^m^−1^ min^−1^), in the absence of IBA57.1. In addition, [4Fe-4S]-NFU4 and [4Fe-4S]-NFU5 are shown to be viable alternative [4Fe-4S]^2+^ cluster donors for ACO2, albeit with slightly lower rates of cluster transfer (second-order rate constants of 1.2 ± 0.3 × 10^4^m^−1^ min^−1^). Hence, our *in vitro* results are consistent with a nonessential role for NFU proteins in plant mitochondria. Moreover, our cluster transfer studies also support mitochondrial aconitase maturation *via* intact [4Fe-4S]^2+^ cluster transfer, rather than a mechanism involving sequential transfer of two [2Fe-2S]^2+^ clusters followed by *in situ* reductive coupling, as recently proposed for human mitochondrial and cytosolic aconitase (57).

**Figure 8:**
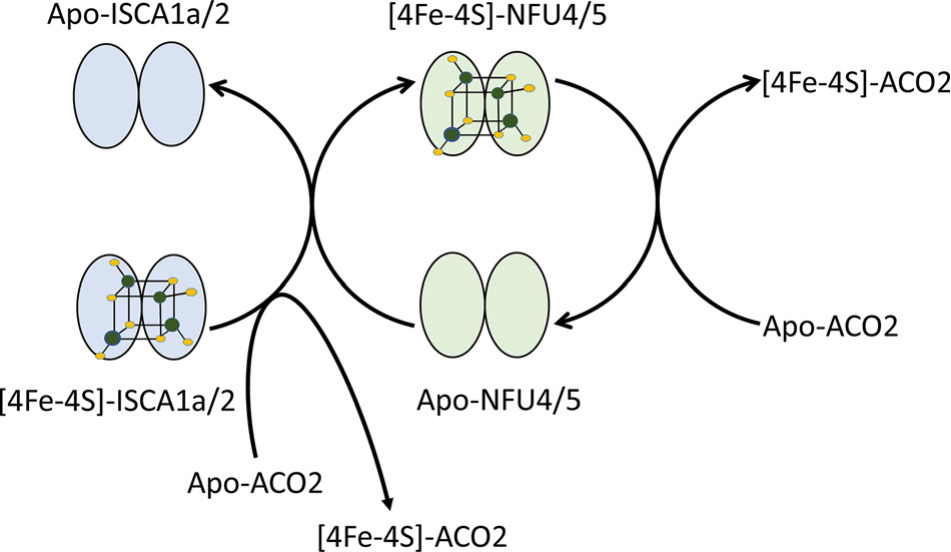
Summary scheme for iron-sulfur cluster trafficking between NFU4 and NFU5 and their partner proteins.

We now have a better picture of the [4Fe-4S] cluster trafficking and interprotein interactions among late-acting ISC components in plants (Fig. 8). The results provide evidence for intact [4Fe-4S] transfer from ISCA1a/2 heterodimer to NFU4 and NFU5 and show that ISCA1a/2 and NFU4/5 are competent for maturation of client [4Fe-4S] cluster-containing proteins. The question of the roles of BOLA and IBA57 remains, as well as the question of the Fe-S cluster donor for INDH.

## Experimental procedures

### Binary yeast two-hybrid assays

Yeast two-hybrid assays were carried out in the Gal4-based yeast two-hybrid reporter strain CY306 (62). The sequences encoding late-acting ISC components devoid of their mitochondrial targeting sequences were cloned into the pGADT7 or pGBKT7 vector (Clontech) between the NdeI or NcoI and BamHI restriction sites (primers used are listed in the Table S1). Gene products result in a protein fused with the Gal4 activation domain or Gal4 DNA-binding domain, respectively. Transformants were selected on yeast nitrogen base medium (0.7% yeast extract w/o amino acids, 2% glucose, and 2% agar) without tryptophan and leucine (−Trp-Leu). Interactions were observed as cells growing on yeast nitrogen base medium in the absence of histidine (−His-Trp-Leu) at 30 °C. The strength of the interactions was evaluated by challenging growth in the presence of 2 or 5 mm of the competitive inhibitor of *HIS3* gene product 3-amino-1,2,4-triazole (3-AT). Images were taken 5 days after dotting (7 µl/dot at an optical density of 0.05 at 600 nm). Results are representative of at least three independent experiments, each on two colonies per transformation event. All constructs producing fusion proteins were also assayed in control experiments after cotransformation with either a pGADT7 or pGBKT7 empty vector.

### Bimolecular fluorescence complementation

The full-length open reading frames coding for ISCAs and NFUs were amplified from *Arabidopsis thaliana* leaf cDNAs with the primers presented in Table S1 and cloned in both pUC-SPYCE and pUC-SPYNE vectors using XbaI and XhoI containing primers (63). The constructs, placed under the control of a CaMV ^35^S promoter, consist of fusions of the proteins of interest at the N terminus of nonfluorescent C- and N-terminal halves of YFP, respectively. *Arabidopsis* protoplasts were prepared and cotransfected with pUC-SPYNE and pUC-SPYCE construct pairs for 5 min in a PEG-based medium as described in (64) without vacuum infiltration. Pairs of constructs involving one empty vector were also transfected as controls to assess if any of the proteins assayed could restore YFP fluorescence in the absence of interacting partners. In prior confocal analyses, fluorescent staining of the mitochondria within cells was performed by incubating freshly transfected protoplasts in a W5 solution (64) containing 100 nm MitoTracker® Orange CMXRos (Invitrogen). The YFP fluorescence in *Arabidopsis* protoplasts was recorded between 520 and 550 nm with an SP8 laser scanning confocal microscope (Leica Microsystems, Wetzler, Germany) after excitation with an argon laser at 514 nm. MitoTracker® Orange CMXRos fluorescence was recorded between 580 and 620 nm after excitation at 560 nm. The Leica LASX software was used to obtain images with and without maximum Z-stack intensity projection. Images were processed using the Adobe Photoshop software package. Results are representative of three independent bombardment experiments including the analysis of 10–20 cells per transformation event.

### Analytical and spectroscopic methods

Protein concentrations were determined by the DC protein assay (Bio-Rad) using BSA (Roche) as standard. Iron concentrations were determined colorimetrically using bathophenanthroline under reducing conditions after digesting proteins in 0.8% KMnO_4_/0.2 m HCl. A calibration curve was constructed from a series of dilutions of a 1000-ppm atomic absorption iron standard.

The preparation and handling of anaerobic samples for spectroscopic studies and cluster transfer experiments were carried out inside a Vacuum Atmospheres glove box under argon atmosphere at an oxygen level of 2 ppm or below. UV-visible absorption spectra were recorded in sealed quartz cuvettes at room temperature using a Shimadzu-3101PC spectrophotometer. CD spectra were recorded in sealed quartz cuvettes using a Jasco J-715 spectropolarimeter. Resonance Raman samples were prepared under strictly anaerobic conditions and comprised 18-µl frozen droplets of protein solutions (∼2 mm in Fe-S clusters) mounted on the cold finger of an Air Products Displex Model CSA-202E closed cycle refrigerator (Air Products and Chemicals, Allentown, PA). Resonance Raman spectra were recorded at 17 K using an Instrument SA Ramanor U1000 scanning spectrometer coupled with a Coherent Sabre argon ion laser. Spectra were recorded by photon counting for 1 s every 0.5 cm^−1^, using 7 cm^−1^ resolution, and each spectrum is the sum of 80–120 scans.

### Cloning, overexpression in E. coli, and purification of recombinant proteins

The cloning of NFU4 (At3g20970) and NFU5 (At1g51390) in pET3d was described previously (65). For cloning in pCDF Duet, ISCA1a (At2g16710) was subcloned from pET28a using the NcoI-BamHI restriction sites of pCDF Duet in which ISCA2 (At5g03905) was cloned in the NdeI-XhoI restriction sites. The *Arabidopsis* ACO2 (At4g26970) sequence was amplified using the primers ACO2 for 5′-CCCCCCCGTCTCCCATGGCTTCTGAGCATTCCTACA-3′ and ACO2 rev 5′-CCCCCTCGAGTTACTTGGCGCTCAAACT-3′ and cloned in the NcoI-XhoI restriction sites of pET15b. Note that a BsmBI type IIs restriction enzyme was used in the ACO2 for primer to generate a compatible NcoI extremity.

The *E. coli* BL21 (DE3) strain was used to coexpress ISCA1a/2 from pCDF-Duet ISCA1a/2 plasmid and the untagged ACO2, which was purified following a procedure described previously for *Azotobacter vinelandii* aconitase (44). The same strain containing the pSBET helper plasmid was used for the expression of untagged NFU4 and NFU5, which were purified aerobically as described previously (65).

### In vitro IscS-mediated Fe-S cluster reconstitution experiments

Apo-ISCA1a/2 was prepared by treating the as-purified [2Fe-2S] cluster-containing ISCA1a/2 with a 50-fold excess of EDTA and a 20-fold excess of potassium ferricyanide under anaerobic conditions and removing the excess reagents by ultrafiltration dialysis using a YM10 membrane. Reconstitutions of Fe-S clusters on as-purified ISCA1a/2, NFU4, NFU5, and ACO2 were carried out under strictly anaerobic conditions. Each recombinant protein was incubated in the presence of a 12-fold excess of FAS, a 12-fold excess of l-cysteine, and a catalytic amount of IscS in the presence of 2 mm DTT for approximately 5 h in the case of ISCA1a/2 heterocomplex, 50 min in the case of NFU4 and NFU5, and 180 min in the case of ACO2. In all cases, the reconstitution mixture was loaded onto a 10-ml Hitrap Q-Sepharose column (GE Healthcare), and the proteins were eluted with an increasing salt gradient of 0–1 m NaCl. A single colored fraction containing predominantly [4Fe-4S]^2+^ cluster-bound ISCA1a/2, NFU4, NFU5, or ACO2 was eluted under an increasing NaCl gradient.

### Fe-S cluster transfer assays monitored by UV-visible CD spectroscopy

The time courses of Fe-S cluster transfer from the cluster-bound donor to apo-acceptor were monitored at room temperature under anaerobic conditions in 1-cm cuvettes using CD spectroscopy. In all cases, the apo-acceptor protein was incubated with 2 mm DTT for 30 min and repurified to remove DTT (DTT pretreatment), prior to initiation of the reaction by the addition of apo-protein to the donor protein solution. The CD spectrum was monitored until no further change was observed. Peek-to-trough or fixed wavelength changes in CD intensity were used to assess the extent of cluster transfer as a function of time. The data were fitted to second-order kinetics using the Kinetiscope chemical kinetics simulator software package (IBM), based on the initial concentration of Fe-S clusters on the donor protein and the concentration of the apo-acceptor protein. The directionality of cluster transfer was assessed by repeating the reaction with cluster-bound acceptor as the donor and the apo-donor as the acceptor. The lability of the donor Fe-S center in the reaction mixture was assessed by monitoring the UV-visible absorption or CD spectrum of the donor, in the absence of the acceptor, over the time course of the reaction. The specific conditions for each cluster transfer reported in this work are given in the results section and in the figure legends.

### Activation of apo-aconitase ACO2 using Fe-S cluster-loaded forms of ISCA1a/2, NFU4, and NFU5

The apo-form of ACO2 was obtained by incubating as-purified ACO2 with EDTA and potassium ferricyanide as described previously (44, 66). Activation mixtures contained 2.4 µm apo*-*ACO2 and [4Fe-4S] cluster-containing ISCA1a/2, NFU4, or NFU5, each 7.4 µm in [4Fe-4S]^2+^ clusters, in 100 mm Tris-HCl, pH 7.8. [4Fe-4S]^2+^ cluster concentrations on ISCA1a/2, NFU4, and NFU5 were assessed based on a molar extinction coefficient at 400 nm of 15.0 mm^−1^ cm^−1^. The same procedure was used in attempts to activate ACO2 (2.4 µm) using [2Fe-2S] cluster-containing ISCA1a/2 (14.5 µm in [2Fe-2S]^2+^ clusters). The [2Fe-2S]^2+^ cluster concentration on ISCA1a/2 was assessed using a molar extinction coefficient at 420 nm of 7.5 mm^−1^ cm^−1^. Activation mixtures were incubated at room temperature under anaerobic conditions, and 10-µl samples were withdrawn at different times and assayed for aconitase activity. Activity was measured spectrophotometrically at 240 nm by following the formation of *cis*-aconitate from citrate or isocitrate, using a molar absorption coefficient ɛ_240_ = 3400 mm^−1^ cm^−1^ for *cis*-aconitate (45, 67). Anaerobically reconstituted samples of ACO2 containing one [4Fe-4S] cluster per protein monomer were used to establish maximal specific activity.

## Data availability

All data are contained within this article and in the supporting information.
