# Supplementary material for: [4Fe-4S] cluster trafficking mediated by Arabidopsis mitochondrial ISCA and NFU proteins
Source: J Biol Chem. 2021 Jan 13;295(52):18367–78. doi: 10.1074/jbc.RA120.015726 (PMC7939391; doi:10.1074/jbc.RA120.015726)
Supplement: Supplementary file 1 [file mmc1.pdf]

Supporting information for the manuscript

[4Fe-4S] cluster trafficking mediated by Arabidopsis mitochondrial ISCA and NFU proteins

Tamanna Azam, Jonathan Przybyla-Toscano, Florence Vignols, Jérémy Couturier, Nicolas Rouhier, and Michael K. Johnson

Figure S1. BiFC negative controls verifying that none of the ISCA proteins tested alone with the empty partner vector can restore YFP fluorescence.

Figure S2. Control studies supporting unidirectional [4Fe-4S] cluster transfer from ISCA1a/2 to NFU4 and NFU5.

Table S1. Primers used in this study.

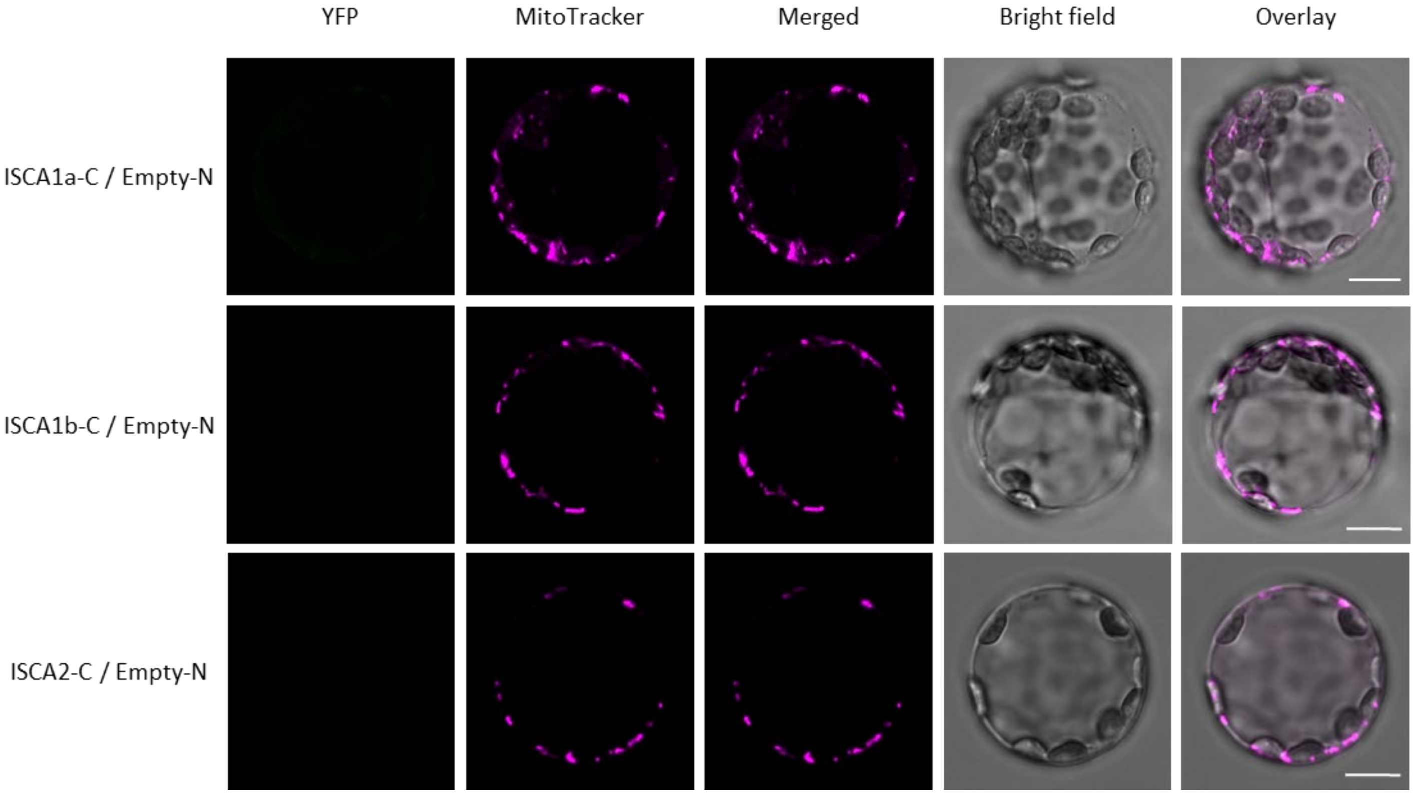

Figure S1. BiFC negative controls verifying that none of the ISCA proteins tested alone with the empty partner vector can restore YFP fluorescence.ISCA-C/empty-N vector pairwises were assayed in Arabidopsis protoplasts isolated from 4 week-old plants in the same conditions as those described for ISCA-C/NFU-N pairwises in Figure 2. Confocal images were captured without Z-stack intensity projection. YFP, yellow fluorescent protein. MitoTracker® Orange CMXRos (Invitrogen) was used at 100 nM to label mitochondria within cells. Bars = 10 µm.

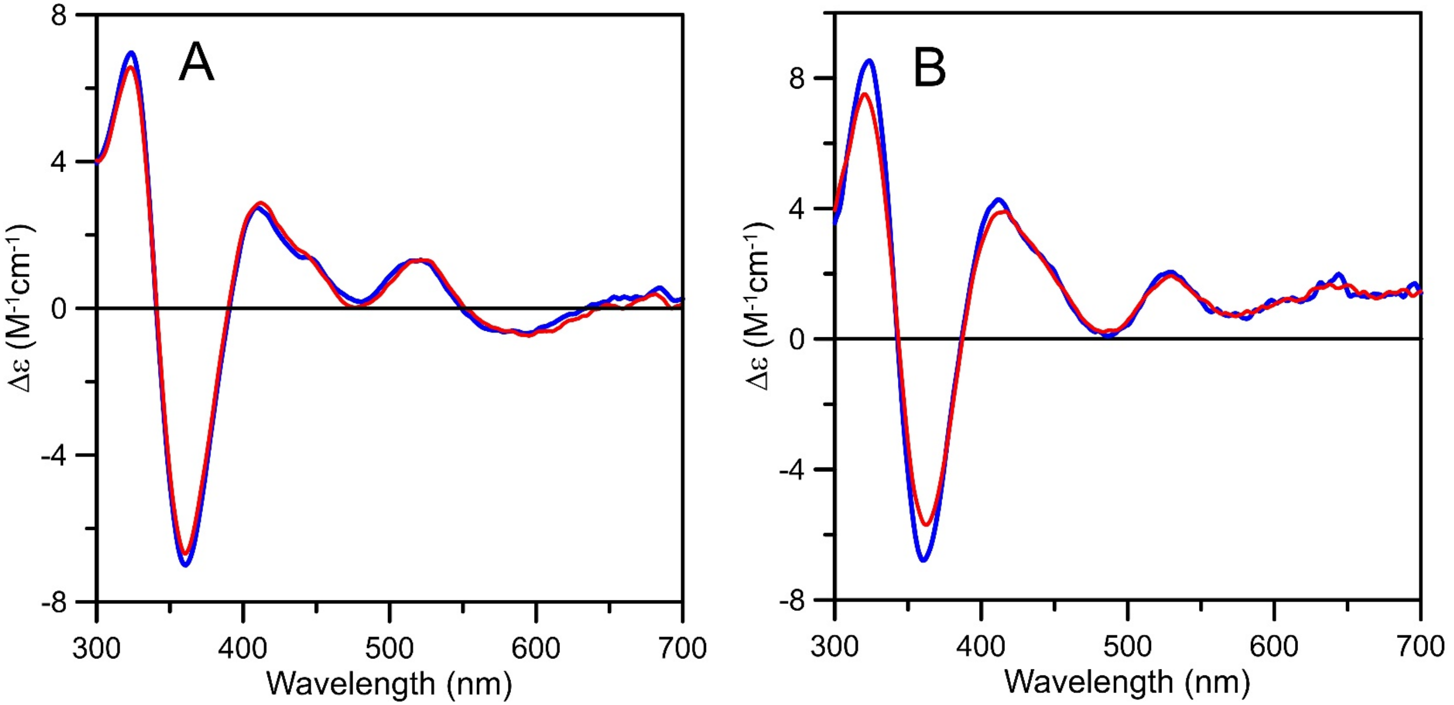

Figure S2. Control studies supporting unidirectional [4Fe-4S] cluster transfer from ISCA1a/2 to NFU4 and NFU5. Attempted [4Fe-4S] cluster transfer from *At* holo-NFU4 (A) and *At* holo-NFU5 (B) to DTT-pretreated *At* apo-ISCA1a/2, monitored by CD spectroscopy as a function of time. CD spectra of the reaction mixture that was initially 50 µM in holo-NFU4 or NFU5 and 80 µM in apo-ISCA1a/2 heterodimers. The blue lines correspond to holo-NFU4 or holo-NFU5 recorded before addition of apo-ISCA1a/2. The red lines correspond to the CD spectra recorded 45 min after the addition of apo-ISCA1a/2. [4Fe-4S] cluster-bound ISCA1a/2 has negligible visible CD, see manuscript text. Consequently, the lack of a significant decrease in the visible CD intensity of [4Fe-4S] cluster-bound NFU4 and NFU5 indicates negligible [4Fe-4S] cluster transfer. The reaction conditions were the same as those used for the successful reverse cluster transfers shown in Figs. 5 and 6.

Table S1. Primers used in this study. Restriction sites required for cloning are in red. For nuclear-encoded plastidial proteins, the sequences coding for the putative targeting sequences have been removed for the cloning in pET and pGADT7/pGBKT7 vectors whereas full-length sequences have been cloned in pUC-SPYNE/SPYCE vectors. Note that a BsmBI type IIs restriction enzyme was used in the ACO2 for primer to generate a compatible NcoI extremity. Primers for cloning GRXS15, BOLA4 in pGADT7/pGBKT7 vectors, are described in (1). The N-terminal domains of NFU4 and NFU5 have been cloned using AtNFU4 for and AtNFU4 rev3 or using AtNFU5 for and AtNFU5 rev3 whereas the C-terminal domains of NFU4 and NFU5 have been cloned using AtNFU4/5 for and AtNFU4 rev or AtNFU5 rev.

**Gene names**	**Plasmids**	**Primer names**	**Sequences**	**N-terminal sequences**
NFU4	pET3d, pGADT7, pGBKT7	AtNFU4 for	5’ CCCCCATGGCTTTTATCCAAACCCAATCA 3’	MAFIQTQS
		AtNFU4 rev	5’ CCCCGGATCCCTACTCTACTCTCATCTC 3’	
	pUC-SPYNE/SPYCE	AtNFU4 for2	5’ CCCCTCTAGAATGAAAGGGATTGCGAGG 3’	MKGIAR
		AtNFU4 rev2	5’ CCCCCTCGAGCTCTACTCTCATCTCTCC 3’	
	pGADT7, pGBKT7	AtNFU4/5 for	5’ CCCCCCCCCATATGGAGGATGACTCTGAAAC 3’	MEDDSE
		AtNFU4 rev3	5’CCCCGGATCCTTAGCCAGAAGAATAGAAATC 3’	
NFU5	pET3d, pGADT7, pGBKT7	AtNFU5 for	5’ CCCCCATGGCTTTTATCCAAACC 3’	MAFIQT
		AtNFU5 rev	5’ CCCCGGATCCTCACTCCATTGGACCAGA 3’	
	pUC-SPYNE/SPYCE	AtNFU5 for2	5’ CCCCTCTAGAATGAAAGGGCTTACGAGG 3’	MKGLTR
		AtNFU5 rev2	5’ CCCCCTCGAGCTCCATTGGACCAGAAGA 3’	
	pGADT7, pGBKT7	AtNFU5 rev3	5’ CCCCGGATCCTTAACCAGAAGAGTAAAAGTC 3’	
ACO2	pET15b	AtACO2 for	5’ CCCCCCCGTCTCCCATGGCTTCTGAGCATTCCTACA 3’	MASEHSY
		AtACO2 rev	5’ CCCCCTCGAGTTACTTGGCGCTCAAACT 3’	
IBA57.1	pGADT7, pGBKT7	AtIBA57.1 for	5’ CCCCCCCCCATATGGCGTCTCGCCTTAAATC 3’	MASRLK
		AtIBA57.1 rev	5’ CCCCGAATTCCTACGCTGCAGCAACTC 3’	
INDH	pGADT7, pGBKT7	AtINDH for	5’ CCCCATATGGCTCTCCGACTACACGGAGTCAAA 3’	MALRLHGVK
		AtINDH rev	5’ CCCGGATCCCTATGATGAGTGGCTAGAATG 3’	
ISCA1a	pET28a, pCDFDUET pGADT7, pGBKT7	AtISCA1a for	5’ CCCCCCCCATATGAAGCAAGTATTAAC 3’	MKQVLT
		AtISCA1a rev	5’ CCCCGGATCCTTAACTAGCACTCTGCTT 3’	
	pUC-SPYNE/SPYCE	AtISCA1a for2	5’ CCCCTCTAGAATGAAAGCTTCTCAAATT3’	MKASQI
		AtISCA1a rev2	5’ CCCCCTCGAGACTAGCACTCTGCTTAGC3’	
ISCA1b	pGADT7, pGBKT7	AtISCA1b for	5’ CCCCCCCCATATGCGAAAGCAAGTATTAGCA 3’	MRKQVLA

		AtISCA1b rev	5’ CCCCGGATCCTCATGTTGTCGTGAATGACTC 3’	
	pUC-SPYNE/SPYCE	AtISCA1bfor2	5’ CCCCTCTAGAATGAGAAAGCAAGTATTA 3’	MRKQVL
		AtISCA1b rev2	5’ CCCCCTCGAGTGTTGTCGTGAATGACTC 3’	
ISCA2	pET12a, pGADT7, pGBKT7	AtISCA2 for	5’ CCCCCCCCATATGTCCCAACCTGAATCG 3’	MSQPES
		AtISCA2 rev	5’ CCCCGGATCCTCAGAGTTTCACCATGAA 3’	
	pCDFDUET	AtISCA2 rev2	5’ CCCCCTCGAGGAGTTTCACCATGAAGGA 3’	
	pUC-SPYNE/SPYCE	AtISCA2 for2	5’ CCCCTCTAGAATGTCAAGATCTCTGGTG 3’	MSRSLV
		AtISCA2 rev3	5’ CCCCCTCGAGGAGTTTCACCATGAAGGA 3’	

Reference

Couturier, J., Wu, H.-C., Dhalleine, T., Pégeot, H., Sudre, D., Gualberto, J. M., Jacquot, J.-P., Gaymard, F., Vignols, F., and Rouhier, N. (2014) Monothiol glutaredoxin-BolA interactions: Redox control of *Arabidopsis thaliana* BolA2 and SufE1. *Mol. Plant* **7**, 187–205
